# A prospective randomized study of efficacy of 2 treatment protocols in preventing recurrence of clinical signs in 51 male cats with obstructive idiopathic cystitis

**DOI:** 10.1111/jvim.15594

**Published:** 2019-08-10

**Authors:** Ran Nivy, Gilad Segev, Dar Rimer, Yaron Bruchim, Itamar Aroch, Michal Mazaki‐Tovi

**Affiliations:** ^1^ Veterinary Teaching Hospital and Koret School of Veterinary Medicine The Hebrew University of Jerusalem Rehovot Israel

**Keywords:** diet, heart rate, hyperkalemia, meloxicam, urinary obstruction

## Abstract

**Background:**

Urethral obstruction (UO) is a common complication of feline idiopathic cystitis (FIC). Robust treatment recommendations to prevent its recurrence are scarce.

**Objectives:**

To evaluate meloxicam treatment for prevention of clinical recrudescence in male cats with obstructive FIC.

**Animals:**

Fifty‐one client‐owned cats.

**Methods:**

Prospective, randomized clinical trial. Every male cat with FIC‐associated UO was deemed eligible for the study and was recruited during hospitalization. After discharge, cats were treated with phenoxybenzamine and alprazolam for 2 weeks, with (24 cats) or without (27 cats) low‐dose meloxicam (0.025 mg/kg/day PO) and monitored for 6 months.

**Results:**

Cumulative number (%) of cats with recurrent UO at 10 days, 1‐, 2‐, and 6‐months after discharge was 1 (2%), 2 (4%), 4 (8%), and 8 (16%), respectively. Overall, 12 (24%) cats experienced signs of recurrent FIC within 6 months, with (8 cats) or without (4 cats) concurrent UO. No difference in the cumulative incidence of UO within 6 months was detected with addition of meloxicam (odds ratio [95% confidence interval], 0.63 [0.13‐2.97]; *P* = .70). All cats were alive at 6 months.

**Conclusions and Clinical Importance:**

No clinical benefit was detected with the addition of low‐dose meloxicam to phenoxybenzamine and alprazolam treatment for 2 weeks after discharge. Nevertheless, this study was underpowered to identify potential differences, and its findings must be corroborated in larger studies.

**Abbreviations**
CKD: chronic kidney disease
COX: cyclooxygenase
FIC: feline idiopathic cystitis
HR: heart rate
IQR: interquartile range
LUT: lower urinary tract
ROC: receiver operator characteristics
rUO: recurrent urethral obstruction
UB: urinary bladder

## 1 INTRODUCTION

Feline idiopathic cystitis (FIC) accounts for up to 55% to 65% of lower urinary tract (LUT) diseases in cats.^1^, ^2^ A plethora of risk factors and purported physiological and behavioral alterations have been associated with FIC, but robust evidence regarding its pathogenesis is lacking, and the underlying causes of this noninfectious, inflammatory disease remain poorly elucidated.^3^ Published risk factors include environmental and husbandry factors, such as indoor lifestyle,^1^, ^4^, ^5^ type of litter,^4^ dry diet,^1^, ^6^ cohabitation with additional cats,^1^, ^4^ conflict with other cats,^7^ and lack of environmental enrichment,^1^, ^3^ in addition to age and obesity.^1^, ^6^, ^7^, ^8^ Neuroendocrine derangements, including increased systemic and localized sympathetic activity vis‐à‐vis suppressed hypothalamic‐pituitary‐adrenal response,^1^, ^3^, ^9^ and increased responsiveness to muscarinic stimulation^3^ have also been implicated in the pathogenesis of FIC. However, barring 1 study, where experimentally induced stress engendered increased urinary bladder (UB) permeability,^9^ direct evidence between stress, abnormal neuroendocrine responses, and UB lesions is lacking.^3^

Irrespective of its etiology, FIC is associated with UB inflammation, manifested by gross cystoscopic changes,^3^, ^10^ increased urinary leukocyte and inflammatory protein concentrations,^11^, ^12^, ^13^ decreased urinary glycosaminoglycan concentration and ulceration, and increased permeability of UB uroepithelium.^1^, ^3^ Additionally, increased urethral pressure and spasm might contribute to clinical signs.^14^

The nebulous pathogenesis of FIC, lack of known etiologies, and its self‐limiting nature preclude tailoring specific, effective treatment.^3^ Only diet regime has been associated with improved outcome and reduction of recurrent episodes in 1 prospective, randomized trial.^15^ Additional treatments, including environmental enrichment,^3^, ^16^ stress reduction,^1^ promoting urethral relaxation,^17^, ^18^, ^19^ and pain management^3^, ^20^ are often advocated, notwithstanding a lack of high‐grade supportive evidence.^3^ Feline pheromones^21^ and glycosaminoglycans,^22^, ^23^, ^24^, ^25^ albeit unsupported by studies, are occasionally administered, especially in refractory recurring cases. Lastly, because FIC is associated with inflammation and pain, and based on current recommendations for idiopathic cystitis management in humans, anti‐inflammatory and analgesic drugs are commonly prescribed to cats, notwithstanding their inefficacy in retrospective and short‐term prospective studies.^17^, ^20^, ^26^ Importantly, most of the aforementioned studies were likely underpowered, and a different outcome might have been found had more cases been recruited.

Although both female and male cats are affected by FIC, in the latter, it might result in urethral obstruction (UO), which might consequently lead to death.^3^ Furthermore, management of obstructed male cats entails urethral catheterization and carries potential complications,^27^ including urethral edema, tear and stricture formation,^27^ anemia,^28^ and catheter‐associated bacteriuria.^29^ Overall, recurrence rate is high, greatly varying between studies and follow‐up periods, ranging from 15% to 65%.^3^, ^6^, ^17^, ^20^, ^30^, ^31^, ^32^ Recurrence and refractory cases might ultimately lead to euthanasia in up to 20% of cases,^3^, ^24^, ^28^, ^31^ underscoring the need for effective long‐term management protocols.

Owing to the consequences of recurrence, and lack of evidence‐based, effective treatment protocols, we aimed to evaluate the efficacy of a 2‐week‐long, low‐dose oral meloxicam treatment in reducing recurrence of urethral obstruction (rUO) in male cats with obstructive FIC. Our primary hypothesis was that low‐dose meloxicam treatment would decrease inflammation during 2 weeks of administration, when FIC was expected to persist, and consequently reduce long‐term recurrence rates. A secondary objective was to investigate possible prognostic markers for recrudescence of clinical signs and the outcome.

## 2 MATERIALS AND METHODS

### 2.1 Cats and definitions

This study was conducted in a referral teaching hospital between the years 2016 and 2018 and was approved by the institutional ethics committee. Male cats with obstructive FIC were prospectively enrolled with their owner's consent. None of the cats had received any medication before admission. Additional inclusion criteria included resolution of azotemia and the ability to fully empty the UB before discharge. Obstructive FIC was diagnosed based on the presence of compatible history, clinical signs, and physical examination findings, including stranguria, hematuria, pollakiuria, periuria, and unsuccessful voiding attempts, in addition to a tense, painful UB, unamenable to manual voiding.^3^ Upon enrollment to the study, abdominal sonography, urinalysis, and urine culture were performed in all cats, whereas survey radiographs and contrast retrograde urethrography were performed in only 20 of 51 cats (39%), due to owners' financial constraints. Nevertheless, all cats that had presented with a recurrent episode during the follow‐up period of this study eventually underwent contrast retrograde urethrography. Data extracted from medical records for statistical analyses included the signalment, season, number of episodes of FIC‐related signs, environment, type of diet before hospitalization and after discharge, clinical signs and duration thereof, vital signs upon admission, weight, duration and type of urinary catheter used during hospitalization and antibiotic treatment.

### 2.2 Collection of samples and laboratory methods

Blood samples for serum chemistry (Cobas 6000; Roche, Mannheim, Germany; at 37°C) and CBC (Advia 2120c, Siemens, Erfurt, Germany) were collected in plain tubes with gel separators and potassium‐EDTA tubes, respectively. In some cases, at the attending clinician's discretion, and depending on the owners' financial constraints, only serum creatinine and electrolytes, PCV, and total solids in plasma (measured by refractometry) were measured. Urine samples (5 mL) were collected by cystocentesis for routine urinalysis and urine culture before urinary catheterization and initiation of fluid therapy.

### 2.3 Treatment and study design

All cats were initially stabilized, and the UO was relieved by catheterization. Medical decisions before relieving the obstruction (eg, type of fluid, administration of IV dextrose, insulin or bicarbonate to control hyperkalemia, medical stabilization before relieving the UO, and the choice of urinary catheter type) and the timing of urinary catheter removal (eg, after azotemia had resolved and urine color and turbidity had improved) were made at the attending clinicians' discretion.

Cats were then randomly assigned to treatment after discharge by drawing the treatment protocol from a sealed envelope to receive either of the following 2 protocols: (1) phenoxybenzamine (Dibenzyline, AX Pharmaceutical Corp, Richmond Hill, Canada; 2 mg/cat, PO, q12h) with alprazolam (Alpralid, CTS Chemical Industries, Kiryat Malachi, Israel; 0.125 mg/cat, PO, q12h) and meloxicam (Loxicom, Norbrook, Newry, Northern Ireland; 0.025 mg/kg, PO, q24h) for 14 days (study group) or (2) phenoxybenzamine and alprazolam alone (control group). In addition, owners were instructed to change the diet to a therapeutic urinary diet and to implement husbandry and environmental measures as previously described.^16^ Cats were subsequently followed up for 6 months. Recurrence of FIC‐related clinical signs and rUO were recorded at 10 days, 1‐, 2‐, and 6‐months after discharge.

### 2.4 Statistical analysis

Fisher's exact test was used to compare the proportion of recurrence between groups (ie, meloxicam treatment, antibiotic treatment, neuter status, breed, season of the year, indoors/outdoors living, type of food [kibble or canned, urinary prescription diet or nonprescription diet], feeding regimen [ad libitum, fixed meals], presence of azotemia, crystalluria, cylinduria, administration of antibiotics during hospitalization and at home, frequency of alprazolam and phenoxybenzamine administration, and type of urinary catheter). Logistic regression was used to determine the association between recurrences and continuous variables (ie, age, weight, number of episode of obstruction, duration of clinical signs, degree of azotemia, serum potassium concentrations, urine specific gravity, degree of proteinuria, urine pH, duration of urinary catheterization and hospitalization, actual dose and duration of alprazolam, phenoxybenzamine treatment). Receiver operator characteristic (ROC) analyses were used to assess the predictive performance of the heart rate (HR) at presentation for the presence of hyperkalemia. Log transformation of continuous variables was used to achieve normal distribution (as determined by the Shapiro‐Wilk test and Q‐Q plots), where appropriate. All tests were 2‐tailed, and *P* < .05 was considered significant. Analyses were done using a statistical software package (SPSS 25.0, IBM, Chicago, Illinois).

Sample size was calculated using an additional statistical software (Gpower 3.0.10). The main outcome of the study for sample size calculation was the effect of treatment. Assuming recurrence rate of 50% in the control group and a risk ratio of 0.2, 19 cats in each group are required to demonstrate a difference with 95% statistical confidence and a power of 80%.

## 3 RESULTS

The study included 51 male cats (intact, 7 cats; neutered, 44 cats), with overall median (interquartile range, IQR) age and body weight of 48 months (24‐72 months) and 6 kg (4.6‐6.5 kg), respectively. The control and study groups included 27 and 24 cats, respectively. Most cats (35; 68%) were domestic short hair. Additional breeds included Persian (4; 8%), Himalayan and British Shorthair (3 each; 6%), Ragdoll and Scottish fold (2 each; 4%), and Siamese and domestic long hair (1 each; 2%). The current obstructive FIC episode was the first in 37 (73%) cats, second in 10 (20%) cats, third in 1 (2%) cat, and fourth in 3 (5%) cats, without differences in their prevalence between treatment groups (*P* > .1). Thirty‐four cats (67%) lived strictly indoors, 2 (4%) lived strictly outdoors, and the remainder (15; 29%) had access to both environments. Before the obstruction, most cats (39; 76%) were fed kibble‐based diet, whereas 11 (22%) were fed both kibble and canned diet. For 1 cat, this information was missing.

At presentation to the hospital, the median (IQR) rectal temperature, HR, and respiratory rate were 38°C (36.7°C‐38.8°C), 180 bpm (132‐200 bpm), and 40 breaths/min (30‐48 breaths/min), respectively. Selected laboratory results at presentation to the hospital are presented in Table 1. Notably, 40 cats (78%) were azotemic (serum creatinine concentration >1.6 mg/dL), and 27 (53%) were hyperkalemic (potassium >5.35 mEq/L) at presentation.

**Table 1:** Selected biochemistry and CBC results from 51 male cats with obstructive feline idiopathic cystitis

Analyte^a^	Median (IQR)		*P* a	Reference interval
	Study group	Control group		
Leukocytes (×10^3^/mL)	15 (13‐21)	15 (11‐23)	.98	6.3‐19.6
Red blood cells (×10^6^/mL)	9 (8‐10)	8 (7‐10)	.28	6.0‐10.1
Platelets (10^3^/μL)	201 (158‐268)	233 (200‐276)	.24	156‐626
Creatinine (mg/dL)	7 (3‐10)	7 (5‐11)	.50	0.6‐1.6
Urea (mg/dL)	162 (73‐267)	228 (119‐410)	.18	38.5‐70.6
Potassium (mEq/L)	5 (4‐7)	6 (5‐8)	.38	3.6‐4.9
Sodium (mmol/L)	147 (141‐150)	145 (142‐150)	.88	151‐158
Chloride (mmol/L)	109 (105‐111)	110 (105‐114)	.72	117‐126

The control group included 27 cats that were treated with phenoxybenzamine and alprazolam for 2 weeks, whereas the study group included 24 cats that received low‐dose meloxicam in addition.

Abbreviation: IQR, interquartile range.

a Comparisons between groups were performed using the Wilcoxon‐Mann‐Whitney test.

Heart rate at presentation had a good predictive performance for hyperkalemia, with an area under the ROC curve of 0.86 (95% confidence interval [CI], 0.74‐0.97). Heart rate cutoff values of 150 and 129 bpm showed specificity of 100% and sensitivity of 50% for hyperkalemia (>5.35 mEq/L) and severe hyperkalemia (>7.0 mEq/L), respectively. Serum potassium concentration and the HR at presentation were significantly associated in a linear regression model, with each 1 mEq increment in serum potassium concentration corresponding to an 18 bpm decrease in HR.

Neither ketamine nor α‐2 adrenergic agonists were used in cats in this study. In addition to fluids administered IV, all cats received during hospitalization phenoxybenzamine (same dose as above) and analgesics (butorphanol, Richter Pharma AG, Wels, Austria; 0.2‐0.3 mg/kg, SC or IV, q4‐6h; or buprenorphine, compounded by the hospital's pharmacy; 0.005‐0.015 mg/kg, IV, q6h‐8 hours), and 33 cats received amoxicillin‐clavulanate (Maclivan, Sandoz GmbH, Kundl, Austria; 15‐20 mg/kg, IV, q12h) pending urine culture results.

Median (IQR) duration of indwelling urinary catheter was 2 (2‐3) days in both groups (*P* = .36), while hospitalization time was 3 (2‐4) days and 3 (3‐5) days in the study and control groups, respectively (*P* = .21). A 5‐Fr, polyvinyl chloride feeding tube (Willy Rusch, Kemen, Germany) or a 4‐Fr, low‐density polyethylene urinary catheter with stylet (Buster cat catheter; Kruuse, Langeskov, Denmark) were used in 41 and 7 cats, respectively, whereas for 3 cats, data were missing. The proportion of cats with either type of urinary catheter did not differ between the study and control groups (*P* = .77).

Median (IQR) phenoxybenzamine dose (administered q12h) was 0.375 mg/kg (0.310‐0.426 mg/kg) versus 0.417 mg/kg (0.345‐0.494 mg/kg) (*P* = .17), and median (IQR) alprazolam dose (administered q12h) was 0.025 mg/kg (0.021‐0.032 mg/kg) versus 0.028 mg/kg (0.023‐0.033 mg/kg) (*P* = .46) in the study and control groups, respectively. After discharge, most cats (42/51) were fed a therapeutic kibble‐based urinary diet (Hill's prescription diet c/d Multicare; 28 cats [55%] and Royal Canin's Urinary care; 14 cats [27%]), and the remainder continued to consume a non‐prescription kibble‐based diet. Eleven cats (22%) that were fed a mixture of kibble and canned food before obstruction continued to consume a non‐prescription canned diet in addition to their kibble diet (either prescription or non‐prescription). Additionally, 28 cats continued antibiotic treatment after discharge for a median of 7 days (range, 1‐14 days), with no difference in the frequency of antibiotic use between treatment groups (*P* = .3).

Overall, recurrent signs of FIC occurred in 12 cats (24%; 6 [25%] cats from the study group and 6 [22%] cats from the control group) over the 6 months after discharge, with (8 cats; 67%) or without (4 cats; 33%) concurrent UO. Recurrent UO occurred in 1, 0, 0, and 2 cats in the study group, and 0, 1, 2, and 2 cats in the control group at 10 days, 1‐, 2‐, and 6‐months after discharge, respectively. Signs of FIC without UO recurred in 3 cats in the study group within 21, 30, and 90 days and in 1 cat in the control group within 6 days. Neither the incidence of rUO or recurrent signs of FIC at any time point after discharge nor their cumulative incidence up to any time point was affected by the addition of meloxicam (*P* > .3; Table 2). With the unexpected low recurrence rate in the control group (22%), the present study with the actual sample size (27 control and 24 study cats) had a power of 30% to reveal a difference, assuming a risk ratio of 5 with 95% confidence.

**Table 2:** Odds ratio for signs of recurrent feline idiopathic cystitis, with or without urethral obstruction, in male cats receiving low‐dose meloxicam treatment for 2‐weeks after discharge, in addition to alprazolam and phenoxybenzamine, compared to cats without meloxicam treatment

	Odds ratio (95% CI)	*P*
Recurrence of obstruction within 10 days	1.04 (0.96‐1.13)	.47
Recurrence of obstruction within 10 days to 1 month	0.96 (0.89‐1.04)	.99
Cumulative recurrence of obstruction within 1 month	1.13 (0.07‐19.12)	.99
Recurrence of obstruction within 1‐2 months	0.92 (0.83‐1.03)	.49
Cumulative recurrence of obstruction within 2 months	0.35 (0.03‐3.59)	.61
Recurrence of obstruction within 2‐6 months	1.05 (0.14‐8.13)	.99
Cumulative recurrence of obstruction within 6 months	0.63 (0.13‐2.97)	.70
Recurrence of signs without obstruction within 6 months	3.5 (0.33‐36.67)	.34

Additionally, duration of indwelling urinary catheter (*P* > .4), presence and severity of azotemia at presentation (*P* > .3), antibiotic administration (*P* > .3), neuter status (*P* > .2), and season of the year (*P* > .4) were not associated with rUO or recurrent signs of FIC at any time point.

No difference in recurrence was detected when cats were fed a prescription, kibble‐based diet, compared to cats that were fed non‐prescription, kibble‐based diet. Similarly, no difference in recurrence rate was observed between cats that were fed a prescription kibble diet, with or without the addition of non‐prescription canned food, to cats that were fed non‐prescription diet (*P* > .3).

All cats survived to discharge and were alive at 6 months after discharge.

## 4 DISCUSSION

In the present randomized, prospective clinical trial, cumulative rUO rates over a 6‐month follow‐up period were low and no differences in recurrence rates at multiple time points were detected with the addition of low‐dose meloxicam. However, the study was underpowered to detect small differences.

Recurrence of clinical signs and UO is common in FIC, might result in life‐threatening complications, and occasionally might lead to euthanasia due to owners' consternation and financial constraints.^3^, ^31^ Barring diet change,^1^, ^15^ treatment protocols to reduce its incidence are scarce, and existing recommendations are often unsubstantiated.^3^ Both treatment protocols used in this study included alprazolam and phenoxybenzamine. The former was administered to reduce FIC‐induced increased urethral pressure,^14^ in addition to its anxiolytic effect, given the association between FIC and stress,^1^, ^9^ whereas the latter was chosen to reduce proximal urethral pressure.^14^, ^19^ The use of α‐1 adrenergic antagonists to reduce proximal urethral pressure is widespread in FIC, due to the findings from urodynamic studies in cats.^17^, ^18^, ^32^, ^33^, ^34^, ^35^ Because increased urethral pressure and anxiety are known causes and consequences of FIC,^1^, ^3^, ^9^, ^14^ all cats herein were administered a benzodiazepine and an α‐1 adrenergic antagonist, whereas meloxicam treatment was randomly assigned only to the study group.

A plethora of evidence supports the inflammatory nature of FIC. Mucosal edema, erythema, ulceration, and prominent blood vessels are noted macroscopically and microscopically,^1^, ^3^, ^36^ whereas histological findings support the notion of chronic inflammation, with increased number of mucosal leukocytes and degranulated mast cells, concomitant increased suburethral proliferation, neovascularization, and elastin content.^37^ Moreover, cats with FIC have increased concentrations of urinary (eg, fibronectin, thioredoxin, total protein)^12^, ^38^ and serum (eg, IL‐12, IL‐18)^11^ pro‐inflammatory proteins. Recently, increased expression of cyclooxygenase (COX)‐1 and COX‐2 in the lamina propria of the UB and urethra was demonstrated in female cats with FIC,^37^ further emphasizing the potential clinical benefit of anti‐inflammatory medications, including nonsteroidal anti‐inflammatory drugs. Notwithstanding the above, several studies failed to demonstrate any clinical benefit of administration of anti‐inflammatory medications in cats with FIC, but these were limited by their retrospective nature, small sample size, and short duration of treatment, as is the current study.^17^, ^20^, ^26^ Similarly, in the present study, a 2‐week low‐dose meloxicam treatment course did not detect a difference in the incidence of relapse. However, several potential limitations must be considered while interpreting the current results. Firstly, with the present low recurrence rate, this study was underpowered to detect potential differences between the groups. Unfortunately, it was unfeasible to predict the proportion of rUO, because previous publications with similar protocols were lacking, whereas others employing different protocols reported significantly higher recurrence rates. Secondly, the meloxicam dose used herein was significantly lower than the licensed dose for cats, namely a single SC injection of 0.3 mg/kg.^39^ There is a paucity of evidence to objectively justify the widespread use of low‐dose (0.02‐0.03 mg/kg/day) meloxicam administration. This treatment regimen has proven safe in long‐term studies, including those involving cats with stable chronic kidney disease (CKD),^40^, ^41^, ^42^, ^43^ and efficacious in improving life quality and alleviating osteoarthritis‐associated pain, as subjectively judged by owners.^40^, ^41^, ^43^ However, only few studies utilized objective measures to demonstrate the efficacy of low‐dose meloxicam treatment,^44^ and in cats with urate‐induced synovitis, low‐dose meloxicam only improved a subjective assessment of pain, whereas higher doses were required to improve objective outcome pain assessment measures.^45^ Furthermore, the inhibitory effects of low‐dose meloxicam on COX‐2 and COX‐1 in cats have not been investigated. Higher doses (0.1 mg/kg) for 7 days were safe in cats with reduced renal mass and stable CKD^46^ and might prove clinically useful in future studies. Importantly, these studies enrolled cats with stable CKD, while cats recovering from UO also recover from potential acute kidney injury. Even though a 5‐day, low‐dose meloxicam treatment in cats with UO was not associated with adverse effects,^20^ the potential deleterious effects of long‐term, extra‐label, higher doses of meloxicam must be considered, and ideally kidney function markers should be monitored. Lastly, the duration of the present treatment might have been too short to document the clinical efficacy of meloxicam. A 2‐week protocol was used herein after previous reports, of considerably shorter duration (≤5 days in total, including hospitalization days), had failed to show clinical benefit,^20^ and because most cases of acute FIC resolve within a week.^3^ Nevertheless, other studies have reported a more protracted course of disease and bladder inflammation,^20^ and recurrence can occur weeks to years after hospitalization, although the highest incidence occurs within the first few months after discharge.^3^, ^20^, ^35^, ^47^ Therefore, a longer treatment course might have resulted in a significant difference between the 2 protocols.

Contrary to 1 study,^6^ and in corroboration with another,^48^ in the present study, the HR at presentation had a good predictive performance for hyperkalemia. Severe hyperkalemia is a life‐threatening condition and an anesthetic risk, warranting immediate medical intervention and postponement of urethral deobstruction under general anesthesia.^49^ Based on the present findings, a 150 bpm HR cutoff should alert clinicians to the presence of hyperkalemia, whereas a 129‐bpm cutoff should prompt immediate medical intervention.

Dearth of information and inconsistent reports also concern dietary recommendations for preventing rUO. In 1, nonrandomized controlled study of a singular diet, a canned formulation proved superior to a dry one in reducing the incidence of rUO,^50^ whereas other retrospective studies failed to demonstrate significant protective effects of diets, canned or dry.^32^, ^51^ The only grade I evidence^52^ treatment recommendation for cats with FIC concerns the consumption of a commercial prescription, cystitis preventive diet, compared to control food, where the former, irrespective of its formulation, reduced the incidence of recurrent signs of FIC.^15^ Although no differences in recurrence rates were detected between the 2 treatment groups, the present study was not designed, and was likely underpowered, to investigate the efficacy of preventive therapeutic diets.

In contrast with previous reports, neither the duration of urinary catheterization^32^ nor the type of urinary catheter^17^ was associated with rUO in our study. Furthermore, both hospitalization‐ and 6‐month case fatality rates were nil, while previously reported case fatality rates of FIC ranged from 5.8% to 8.9%,^6^, ^31^, ^53^, ^54^ and none of our cats was euthanized, notwithstanding a similar recurrence rate to previous studies.^3^, ^6^, ^17^, ^20^, ^30^, ^31^, ^32^ Recurrence is purportedly a leading cause of euthanasia in FIC, leading to euthanasia of 21% of cats in 1 study.^31^ The discrepancy between the present study and previous ones likely reflects differing attitudes among clinicians, owners, and institutions (eg, reluctance to euthanize and financial aid funds) and, possibly, improved critical case management.

In addition to the above‐outlined limitations regarding cohort size, meloxicam dosing, and duration of treatment, this study had several additional limitations, which undermined standardization between the 2 treatment groups. Firstly, although all cat owners had been instructed to feed their cats with specific therapeutic urinary diets, different brands were administered, and occasionally, canned, commercial, non‐prescription diets were added. Secondly, the addition of antimicrobial treatment was at the attending clinicians' discretion, against current recommendation guidelines^55^ and was always done in the absence of supporting bacteriological evidence. Although antimicrobial treatment in FIC has not been associated with improved outcome or decreased incidence of rUO,^17^ it might have affected the present results nonetheless. Thirdly, there were several confounding factors that were not standardized among cats, including diet and implementation of environmental enrichment techniques, which might have affected results. Additionally, urethral deobstruction was performed by different clinicians with variable experience, which might have affected the chances of rUO. In many instances, contrast retrograde urethrography was not performed upon first admission due to owners' financial constraints. However, all cats that had sustained a recurrent FIC episode had eventually undergone LUT contrast studies, and urethral strictures and calculi were therefore ruled out. Additionally, although the study was prospective in nature, some data were retrospectively retrieved from medical records (eg, rectal temperature, urinary catheter type) and were thus missing in a minority of cases. Lastly, the study was performed at a university referral hospital and, based on our post hoc power analysis, was underpowered, rendering generalization to larger populations of cats difficult.

In conclusion, both protocols used in the present study were associated with relatively low rUO rates over the 6‐month follow‐up period, but future studies are warranted to investigate the clinical utility of meloxicam treatment in decreasing the incidence of recurrent signs of FIC in a larger cohort of cats and possibly at higher doses and for a longer duration.

## CONFLICT OF INTEREST DECLARATION

Authors declare no conflict of interest.

## OFF‐LABEL ANTIMICROBIAL DECLARATION

Authors declare no off‐label use of antimicrobials.

## INSTITUTIONAL ANIMAL CARE AND USE COMMITTEE (IACUC) OR OTHER APPROVAL DECLARATION

The study was approved by the institutional ethics committee (reference number: KSVM_VTH/24_2017).

## HUMAN ETHICS APPROVAL DECLARATION

Authors declare human ethics approval was not needed for this study.
